# Melatonin Promotes Superovulation in Sika Deer *(Cervus nippon)*

**DOI:** 10.3390/ijms150712107

**Published:** 2014-07-08

**Authors:** Liang Wang, Zhi-Yong Zhuo, Wen-Qing Shi, Dun-Xian Tan, Chao Gao, Xiu-Zhi Tian, Lu Zhang, Guang-Bin Zhou, Shi-En Zhu, Peng Yun, Guo-Shi Liu

**Affiliations:** 1 Key Laboratory of Animal Genetics and Breeding of the Ministry of Agriculture, National Engineering Laboratory for Animal Breeding, College of Animal Science and Technology, China Agricultural University, Beijing 100091, China; E-Mails: wangliangcau@139.com (L.W.); zzy_8702@163.com (Z.-Y.Z.); gaochaowby@gmail.com (C.G.); tian7550@163.com (X.-Z.T.); 1233456zhanglu@sina.com (L.Z.), zhushien@cau.edu.cn (S.-E.Z.); 2 Animal Husbandry Station of Beijing, Beijing 100101, China; E-Mail: shiwenqing2003@126.com; 3 Department of Cellular & Structural Biology, the UT Health Science Center, San Antonio, TX 78229, USA; E-Mail: Tan@uthscsa.edu; 4 Institute of Animal Genetics and Breeding, College of Animal Science and Technology, Sichuan Agricultural University (Chengdu Campus), Chengdu 611130, China; E-Mail: zguangbin@sicau.edu.cn

**Keywords:** melatonin, superovulation, FSH, LH, PRL, sika deer

## Abstract

In this study, the effects of melatonin (MT) on superovulation and reproductive hormones (melatonin, follicle-stimulating hormone (FSH), luteinizing hormone (LH) and PRL) were investigated in female sika deer. Different doses (40 or 80 mg/animal) of melatonin were subcutaneously implanted into deer before the breeding season. Exogenous melatonin administration significantly elevated the serum FSH levels at the time of insemination compared with levels in control animals. During superovulation, the serum LH levels in donor sika deer reached their highest values (7.1 ± 2.04 ng/mL) at the point of insemination, compared with the baseline levels (4.98 ± 0.07 ng/mL) in control animals. This high level of LH was sustained until the day of embryo recovery. In contrast, the serum levels of PRL in the 80 mg of melatonin-treated group were significantly lower than those of control deer. The average number of corpora lutea in melatonin-treated deer was significantly higher than that of the control (*p* < 0.05). The average number of embryos in the deer treated with 40 mg of melatonin was higher than that of the control; however, this increase did not reach significant difference (*p* > 0.05), which may be related to the relatively small sample size. In addition, embryonic development in melatonin-treated groups was delayed.

## 1. Introduction

Superovulation technology has been widely applied in assisted reproductive technologies of livestock and economic animals. However, the superovulation efficiency of sika deer (*Cervus nippon*) is considerably lower than that of other species [1,2], which limits the progress of sika deer farming. In a previous study, we found that the administration of melatonin implants could improve the number of corpora luteum and recovered embryos in sheep [3]. Therefore, we expect that melatonin implantation may also promote superovulation in sika deer. 

Melatonin is a derivative of the essential amino acid, tryptophan, and possesses multiple physiological functions. Melatonin application is widely used to improve reproductive performance in several species [4,5,6]. In short-day breeders, melatonin stimulates the release of gonadotropin-releasing hormone (GnRH) from the hypothalamus-pituitary-gonadal (HPG) axis, thereby regulating pituitary secretion of luteinizing hormone (LH) and follicle-stimulating hormone (FSH) to control estrus and ovulation [7,8].

There are few reports related to the reproductive physiology of sika deer, including the profiles of their reproductive hormone during the breeding season. Thus, the purpose of this study was to investigate our hypothesis that melatonin implants can alter the levels of reproductive hormones (FSH, LH and PRL), thereby promoting the superovulation effect., whereas this is the first observation of the effects of melatonin on superovulation and reproductive hormones of the rare species (sika deer), which has unique reproductive characteristic different from domestic animals. Although this study was partially based on the results of our previous report [1,6,9,10], we firstly report the LH and FSH increase after melatonin treatment in deer. This improved superovulation and the number of useful embryos.

## 2. Results

### 2.1. The Concentration of MT, FSH, LH and PRL in Sika Deer after MT Implantation

#### 2.1.1. Melatonin Concentrations

**Figure 1:**
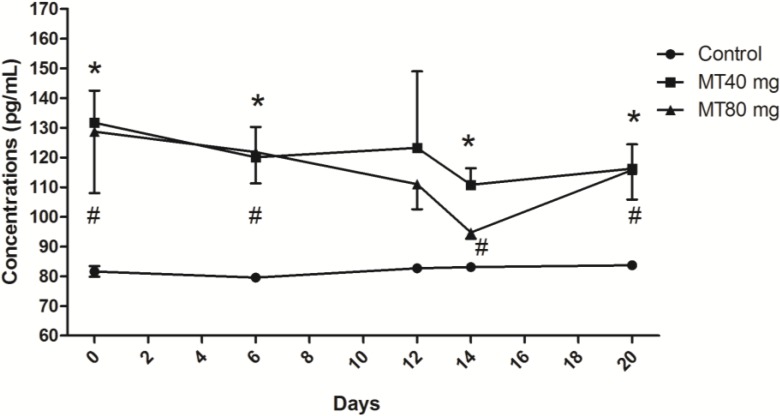
Melatonin concentrations throughout the study period. Day 0: controlled intravaginal drug release (CIDR) insertion; Day 6: CIDR substitution; Day 12: CIDR removal; Day 14: insemination; Day 20: embryo recovery. In two melatonin-treated groups, the melatonin levels dropped after CIDR removal; however, these levels were still significantly higher than those of the control (*p* < 0.05). The decreased melatonin levels rose again during the period between the insemination and embryo recovery. In the control group, the melatonin levels did not change among various stages. Values are given as the mean ± S.E.M. The error bar is extended above and below at each time point in the MT40 mg group and MT80 mg group, respectively. “*” or “#” mean *p* < 0.05.

The melatonin levels in two melatonin-treated groups were similar at the various stages of superovulation and dropped the first time after controlled intravaginal drug release (CIDR) removal; however, these levels remained significantly higher than those of the control animals (*p* < 0.05) (Figure 1). This indicated that the subcutaneously implanted melatonin was constantly released into the blood circulation. Interestingly, after CIDR removal, the decreased melatonin levels rose again during the period between the insemination and embryo recovery in both of the melatonin-treated groups; this phenomenon was not observed in control animals (Figure 1). In the control group, the melatonin levels did not change among various stages (Figure 1).

#### 2.1.2. FSH Concentrations

**Figure 2:**
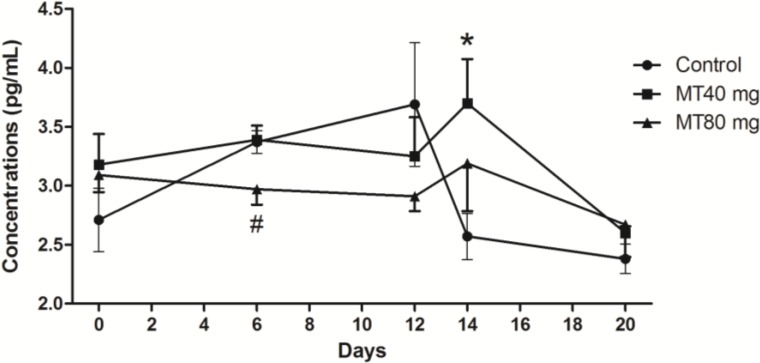
FSH concentrations throughout the study period. Day 0: CIDR insertion; Day 6: CIDR substitution; Day 12: CIDR removal; Day 14, insemination; Day 20: embryo recovery. During Days 12 to 14, the FSH concentrations in the control group dropped from their peak values to baseline levels; however, in the melatonin-treated deer, the FSH concentrations rose from their baseline levels to their peak values. These peak values were maintained only for a short time and decreased to their baseline levels again. Values are given as the mean ± S.E.M. The error bar is extended above and below at each time point in the MT40 mg group and MT80 mg group, respectively. “*” or “#” mean *p* < 0.05.

Significant changes in FSH were observed on Days 12 and 14 during the superovulation process (*p* < 0.05). During this period, FSH concentrations in the control group dropped from their peak values to baseline levels; however, the inverse phenomenon was observed in the melatonin-treated deer. That is, the FSH concentrations rose from their baseline levels to their peak values (Figure 2). These peak values were maintained only for a short time and rapidly decreased to their baseline levels again. 

#### 2.1.3. LH Concentrations

**Figure 3:**
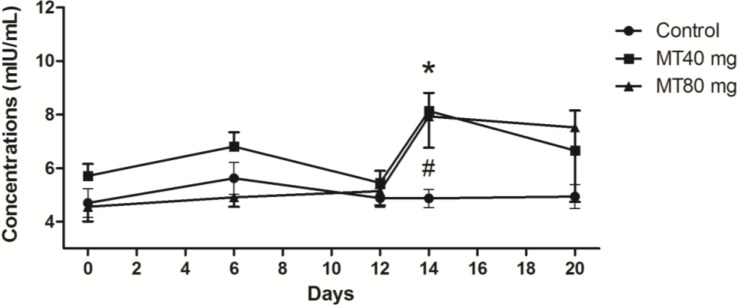
LH concentrations throughout the study period. Day 0: CIDR insertion; Day 6: CIDR substitution; Day 12: CIDR removal; Day 14: insemination; Day 20: embryo recovery. In melatonin-treated animals, the highest LH values were observed on Day 14, while in the control group, LH levels were stable during the process. Values are given as the mean ± S.E.M. The error bar is extended above and below at each time point in the MT40 mg group and MT80 mg group, respectively. “*” or “#” mean *p* < 0.05.

LH levels in the control group were stable during the process of superovulation. Significant fluctuations of LH occurred in melatonin-treated animals (*p* < 0.05). The highest LH values in melatonin-treated animals were observed on the insemination day (Day 14) of superovulation; the high levels of LH persisted until the day of embryo recovery (Figure 3).

#### 2.1.4. PRL Concentration

**Figure 4:**
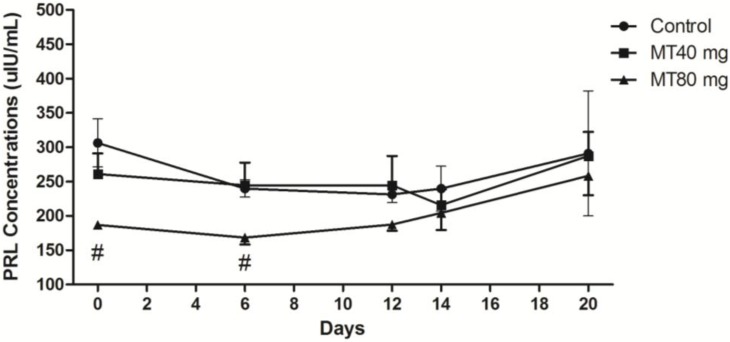
PRL concentrations throughout the study period. Day 0: CIDR insertion; Day 6: CIDR substitution; Day 12: CIDR removal; Day 14: insemination; Day 20: embryo recovery. The PRL levels in the 80 mg melatonin-treated animals were significantly elevated compared with those of the other animals during the entire process. Values are given as the mean ± S.E.M. The error bar is extended above and below at each time point in the MT40 mg group and MT80 mg group, respectively. “#” mean *p* < 0.05.

The data indicated that after melatonin implantation, the PRL levels in all groups slowly decreased. After CIDR removal, the PRL levels rose again (Figure 4). The PRL levels in the high-dose (80 mg/animal) melatonin-treated animals were significantly elevated compared with those of the other animals during the entire process of superovulation (*p* < 0.05) (Figure 4).

### 2.2. Correlation between the Levels of Melatonin and FSH, LH and PRL

Several strong correlations were observed between the serum levels of melatonin and FSH, LH and PRL at various stages of superovulation. The data, which were derived from Figure 1, Figure 2, Figure 3 and Figure 4, are listed in Table 1.

**Table 1:** The correlation coefficient of MT and FSH, LH and PRL.

Blood Collection Stage	Hormone		Control Group	Group 1 (40 mg Implants)	Group 2 (80 mg Implants)
CIDR insertion (Day 0)	MT	FSH	−0.629	−0.494	0.912
		LH	−0.458	0.940	0.996 *
		PRL	0.854	0.464	0.659
CIDR substitution (Day 6)	MT	FSH	−0.738	0.829	0.318
		LH	−0.863	0.529	−0.074
		PRL	0.794	0.567	−0.169
CIDR Removal (Day 12)	MT	FSH	−0.666	0.086	−0.043
		LH	−0.105	−0.056	0.991 *
		PRL	0.887	−0.706	−0.043
Insemination (Day 14)	MT	FSH	−0.246	−0.281	0.178
		LH	−0.179	−0.832	0.432
		PRL	0.293	−0.855	0.311
Embryo Recovery (Day 20)	MT	FSH	−0.103	−0.960 *	0.851
		LH	−0.095	−0.268	−0.384
		PRL	0.076	−0.644	−0.313

* Strong correlations *p* < 0.05.

### 2.3. Effects of Melatonin Implantation on Superovulation in Female Sika Deer

**Figure 5:**
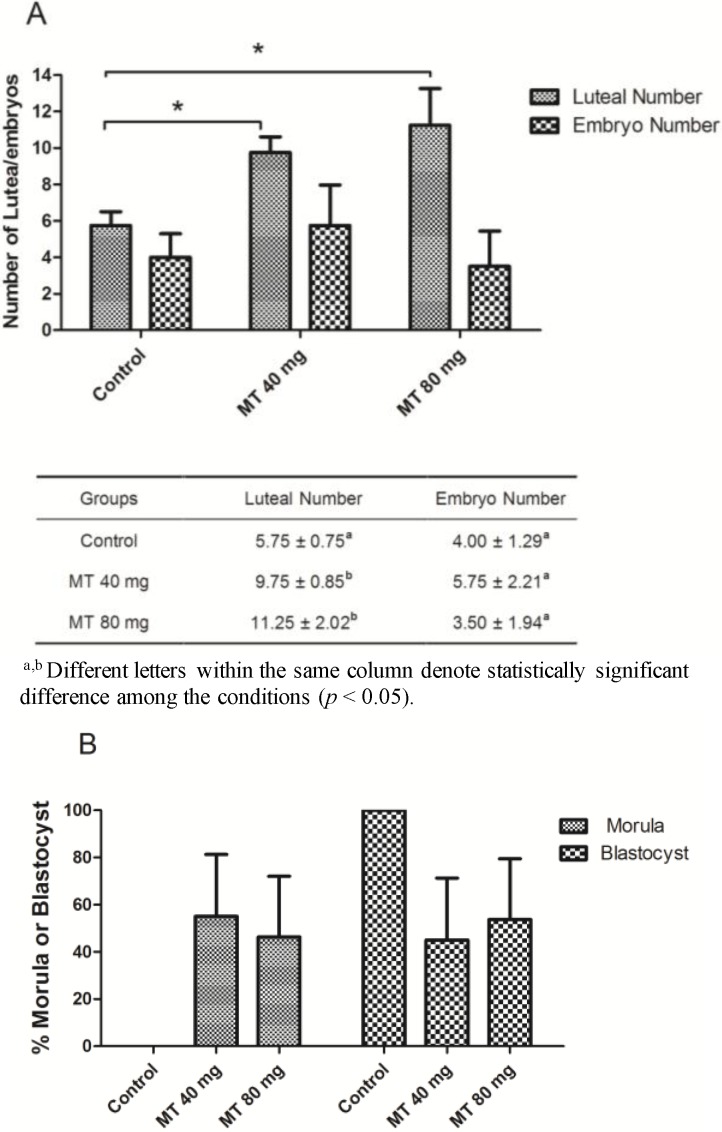
(**A**) The number of corpora lutea and embryos; (**B**) embryonic development stage. All embryos developed into the blastocyst stage in the control animals, while nearly half of the embryos from the melatonin-treated animals remained in the morula stage, although this phenomenon failed to exhibit significant differences. “*” mean *p* < 0.05.

The results revealed that the numbers of corpora lutea in the melatonin-implanted animals were significantly higher than those of the control animals (*p* < 0.05) (Figure 5A). With respect to the number of embryos recovered, there was an increased tendency for embryo recovery in the 40-mg melatonin-implanted animals compared with the control group. However, this increase did not reach statistical significance (*p* > 0.05).

It appears that embryonic development in the melatonin-treated groups was delayed. In the control animals, all embryos developed into the blastocyst stage. However, nearly half of the embryos from the melatonin-treated animals remained in the morula stage, although this phenomenon failed to exhibit significant differences, due to the shortage of recovered embryos (*p* > 0.05) (Figure 5B).

## 3. Discussion

The photoperiod regulates estrus by affecting the secretion of reproductive hormones, which are regulated by the serum levels of melatonin released from pineal gland [11]. To test the effects of melatonin on reproductive performance, melatonin was implanted into sika does during the process of superovulation. This implanted melatonin significantly increased serum melatonin levels. Accordingly, the profiles of several reproductive hormones, including FSH, LH and PRL, were also altered under the influences of the elevated melatonin levels [3,12,13]. Increased levels of LH and FSH in porcine prenatal follicles have been reported to improve the quality of oocytes [14]. This implies that melatonin implantation resulted in the improvement of doe superovulation. Once the CIDRs were removed at Day 12 of superovulation, the serum melatonin levels in the treated animals rapidly dropped to their lowest point on the day of insemination (Day 14), at which point, the follicle development gradually increased and resulted in ovulation. This observation is consistent with a previous report [15] in which the authors found that reduced melatonin levels were associated with an increase in the diameter of the follicles. Melatonin levels are higher in follicles than in peripheral serum before ovulation in humans [16,17], which indicates that the ovarian function can be directly affected by melatonin, as well as indirectly affected via the hypothalamus-pituitary axis. We speculate that the accumulated melatonin in the follicles promotes their development.

Melatonin stimulates the release of GnRH through the HPG axis, thereby regulating pituitary secretion of LH and FSH. Melatonin has been suggested to partially suppress the hypothalamus-pituitary-ovarian axis and to induce differential regulation of sex steroid receptors in the ovary, oviduct and uterus during ovulation [13]. Melatonin is reported to promote the synthesis and secretion of FSH, LH and PRL directly by either the hypothalamus or by β-adrenergic receptors localized in ovaries or adrenal cells [18,19,20,21]. However, the impact of melatonin on pituitary activity is mediated via a negative feedback through estradiol [21]. High levels of melatonin receptors have been found in the pars tuberalis of the pituitary in mammals [22]. By binding to the subtypes of receptors, melatonin exhibits dual roles (promotes or inhibit) on the secretion of reproductive hormones under different physiological conditions or in different species [23].

Our results demonstrate that after an injection of FSH on Day 9 at the time of superovulation, the FSH concentrations in control group were elevated, followed by a reduction after CIDR withdrawal. In contrast, in the presence of melatonin, the FSH levels were elevated only after CIDR withdrawal. It seems that the FSH peak values in the melatonin-treated groups were delayed under the residual action of melatonin compared with that of control animals. These results indicated that without FSH intramuscular injection, the concentrations of FSH rose rapidly and persisted till to insemination in the treated groups, even in the absence melatonin. As a result, during the period of CIDR withdrawal to insemination, the release of FSH was associated with decreased concentrations of melatonin, which suggested that melatonin participated in the regulatory mechanism of FSH production in the serum. 

It appears that the reduced melatonin concentrations between the time of CIDR removal until insemination was critical for the promotion of LH release. This resulted in a peak level of LH at the time of insemination, which promotes ovulation (Figure 3). As LH muscular injection occurred at the time of insemination, the elevated LH levels could not be explained by exogenously applied LH.

Many studies have reported that melatonin influences PRL release in mammals [24,25,26]. Melatonin inhibits PRL gene expression either through the inhibition of thyrotropin-releasing hormone (TRH), which upregulates PRL expression, or by direct inhibition of the anterior pituitary [24]. It has been reported that 21 days after melatonin treatment, PRL concentrations are at their lowest levels. In addition, estrus in non-pregnant does and lactating does advances approximately 35 and 38 days, respectively [27]. PRL concentrations after high doses of implanted melatonin were significantly suppressed compared with those in the other groups (Figure 4). The low levels of PRL contribute to doe estrus and ovulation and verify the inhibitory effect of melatonin on PRL.

The seasonal serum melatonin variations influence the follicular maturation process, which, in turn, affects the secretion of reproductive hormones. It was reported that the melatonin concentrations in human preovulatory follicles are several-fold higher than those in the serum [16,17]. This indicated that melatonin may directly affect ovarian function. Melatonin concentrations in mature follicular fluid *in vivo* were significantly higher than those of mature follicles cultured *in vitro* [28]. According to the analysis of the current study, there are strong correlations between MT and FSH, LH and PRL. In particular, at low doses of melatonin, melatonin and FSH levels exhibit a significantly negative correlation at the time of embryo recovery. Furthermore, there was an extremely strong positive correlation between melatonin and LH levels at the highest melatonin dose (Table 1). It is interesting that a negative correlation between melatonin and FSH levels was observed in control does. However, this correlation became positive after melatonin implantation in treated does and exhibited a dose response. That is, the more melatonin that was implanted, the stronger the correlation (Table 1). The results indicated that before estrus, high levels of melatonin caused by melatonin implantation promoted LH release. The negative correlation suggests that during ovulation, melatonin is actively transported into the follicles, which leads to superovulation. The negative correlation between melatonin and PRL levels proved that melatonin promoted estrus by inhibiting PRL release.

Melatonin has beneficial effects on embryonic development in old female goats under superovulation [4]. Encouraged by this result, we planned to increase the odds of success in embryo transplantation with melatonin implantation in sika deer. To reach this goal, the first step is to increase the quality of superovulation. However, there are few reports related to the effect of melatonin on superovulation. In the present study, we found that melatonin implantation prolonged FSH release, promoted the release of LH and inhibited PRL in sika deer. These changes led to improved superovulation. The changes of FSH and LH in melatonin-treated female sika deer may result in the delay of ovulation and, thereby, postpone embryonic development. Most importantly, we, for the first time, reported that both doses (40 and 80 mg) of melatonin significantly increased the numbers of corpora lutea in recipient deer compared with the control group. In addition, the 40 mg of melatonin implantation exhibited a tendency towards better embryo recovery than that in the control group, although this tendency failed to achieve statistical significance. This may be related to the sample size involved. We realize that the limitation of this study is the sample size of animals (four sika deer/group). This obstacle is difficult to be overcome, due to the limited availability of these large animals. However, this study provides valuable preliminary information on the use of melatonin to improve superovulation. The results obtained from the current study provide first-hand evidence for the use of melatonin to increase reproductive efficiency in sika deer, a rare species.

## 4. Experimental Section

### 4.1. Materials

Twelve female sika deer donors were selected and bred at the Beijing Lushen Deer Industry Co., Ltd. (Beijing, China), deer breeding farm. All experimental protocols regarding the handling of sika deer were in accordance with the requirements of the Institutional Animal Care and Use Committee at China Agricultural University.

### 4.2. Methods

#### 4.2.1. Melatonin Implantation

Multiparous weaned hinds (*N* = 8) were selected on 18 July 2008, before the breeding season, 80 days before estrous synchronization. Melatonin was subcutaneously implanted (subcutaneous sustained release melatonin, a cylindrical implant, containing 10 mg MT, which was made in our lab) at the root of the right ear. Female sika deer in Treatment 1 (*N* = 4) each received 40 mg of melatonin implants, and female sika deer in Treatment 2 (*N* = 4) each received 80 mg of melatonin implants. Female sika deer in Treatment 3 (*N* = 4) served as the controls and received no melatonin treatment.

#### 4.2.2. Estrous Synchronization, Superovulation, Insemination and Embryo Recovery

Estrous synchronization, superovulation, insemination (frozen semen from a single buck) and embryo recovery were performed as described previously [1]. Briefly, estrous synchronization treatment consisted of an ovine CIDR (InterAg, Hamilton, New Zealand). The date of CIDR insertion was designated Day 0 (initial day of treatment). Superovulation was conducted with a total dose of 320 mg of Folltropin-V (Bioniche, Belleville, ON, Canada). Intramuscular injections were given every 12 h (40 mg for each injection), beginning on the afternoon of Day 9. The CIDRs were removed on Day 12, concurrent with the seventh injection of FSH. Insemination was performed (transcervical technique) 2 days after CIDR removal, with insemination repeated 8 to 12 h later. Embryos were surgically collected 6 days after the first insemination.

#### 4.2.3. Blood Collection

Blood was collected on the right side of the carotid at the time of CIDR insertion, CIDR substitution, CIDR removal, insemination and embryo recovery during superovulation from 20 September to 10 October.

#### 4.2.4. Preparation of Serum

Blood was inverted for 2 to 4 h at room temperature, and the blood clots were stripped with sterile needles. The tubes were centrifuged at 3000× *g*, 4 °C for 10 min, and the sera were separated. The sera were carefully collected into 1.5-mL centrifuge tubes (one for testing and one spare) and stored at −20 °C until the assays were performed.

#### 4.2.5. Assay for Hormone Concentrations

MT levels in serum samples were quantified using an enzyme-linked immunosorbent assay (ELISA) kit (MT ELISA kit, Mybiosource, Inc., San Diego, CA, USA). In this ELISA assay, the detection range of MT was from 6.25 to 400 pg/mL. The coefficients of variation (CV) were 7.53% and 11.3% for intra-assay and inter-assay variability, respectively. 

LH, FSH and PRL levels in serum samples were measured by a commercial radioimmunoassay (RIA) kit (Beijing North Biotechnology Institute, Beijing, China) using a gamma counter. The coefficients of variation (CV) were 10% and 15.8% (FSH), 2.0% to 2.4%, 4.2% to 7.5% (LH) and 10% and 15.7% (PRL) for intra-assay and inter-assay variability, respectively. The recovery rates were 94% to 105% (FSH), 90% to 105% (LH) and 90% to 109% (PRL). The sensitivity of the assay was 1.0 mIU/mL for both FSH and LH, and the detection range of PRL was 125–2000 µIU/mL (PRL), respectively.

### 4.3. Statistical Analysis

All data are expressed as the mean ± S.E.M. Data were subjected to multiple comparison analysis using one-way ANOVA analysis for intergroup comparison with SPSS 18.0 statistical software, and the correlations were analyzed using the correlations procedure. *p* < 0.05 was considered statistically significant.

## 5. Conclusions

In this study, for the first time, we reported that exogenous melatonin implantation into female sika deer modified the profiles of their reproductive hormones and improved their superovulation, as demonstrated by an increase in the number of corpora lutea and embryos recovered. The current observations provide valuable information regarding the use of melatonin to increase reproductive efficiency in female sika deer.

## References

[1] Wang L., Zhou G.B., Shi W.Q., Shi J.M., Tian X.Z., Gao C., Zhang L., Zhu S.E., Zhang T.T., Zeng S.M. (2012). First live offspring born in superovulated sika deer (*Cervus nippon*) after embryo vitrification. Theriogenology.

[2] Soler J.P., Mucci N., Kaiser G.G., Aller J., Hunter J.W., Dixon T.E., Alberio R.H. (2007). Multiple ovulation and embryo transfer with fresh, frozen and vitrified red deer (*Cervus elaphus*) embryos in Argentina. Anim. Reprod. Sci..

[3] Zhang L., Chai M., Tian X., Wang F., Fu Y., He C., Deng S., Lian Z., Feng J., Tan D.X. (2013). Effects of melatonin on superovulation and transgenic embryo transplantation in small-tailed han sheep (Ovis aries). Neuro Endocrinol. Lett..

[4] Forcada F., Abecia J.A., Cebrian-Perez J.A., Muino-Blanco T., Valares J.A., Palacin I., Casao A. (2006). The effect of melatonin implants during the seasonal anestrus on embryo production after superovulation in aged high-prolificacy Rasa Aragonesa ewes. Theriogenology.

[5] Barrett P., Bolborea M. (2012). Molecular pathways involved in seasonal body weight and reproductive responses governed by melatonin. J. Pineal Res..

[6] Gao C., Han H.-B., Tian X.-Z., Tan D.-X., Wang L., Zhou G.-B., Zhu S.-E., Liu G.-S. (2012). Melatonin promotes embryonic development and reduces reactive oxygen species in vitrified mouse 2-cell embryos. J. Pineal Res..

[7] Malpaux B., Migaud M., Tricoire H., Chemineau P. (2001). Biology of mammalian photoperiodism and the critical role of the pineal gland and melatonin. J. Biol. Rhythms.

[8] Reiter R.J., Tan D.-X., Manchester L.C., Paredes S.D., Mayo J.C., Sainz R.M. (2009). Melatonin and Reproduction Revisited. Biol. Reprod..

[9] Wang F., Tian X., Zhang L., Tan D., Reiter R.J., Liu G. (2013). Melatonin promotes the *in vitro* development of pronuclear embryos and increases the efficiency of blastocyst implantation in murine. J. Pineal Res..

[10] Wang F., Tian X., Zhang L., Gao C., He C., Fu Y., Ji P., Li Y., Li N., Liu G. (2014). Beneficial effects of melatonin on *in vitro* bovine embryonic development are mediated by melatonin receptor 1. J. Pineal Res..

[11] Malpaux B., Thiéry J.-C., Chemineau P. (1999). Melatonin and the seasonal control of reproduction. Reprod. Nutr. Dev..

[12] Bubenik G., Smith P., Schams D. (1986). The effect of orally administered melatonin on the seasonality of deer pelage exchange, antler development, LH, FSH, prolactin, testosterone, T3, T4, cortisol, and alkaline phosphatase. J. Pineal Res..

[13] Chuffa L.G.A., Seiva F.R., Fávaro W.J., Teixeira G.R., Amorim J.P., Mendes L.O., Fioruci B.A., Pinheiro P.F.F., Fernandes A.A.H., Franci J.A. (2011). Melatonin reduces LH, 17 beta-estradiol and induces differential regulation of sex steroid receptors in reproductive tissues during rat ovulation. Reprod. Biol. Endocrinol..

[14] Wu J., Xu B., Wang W. (2007). Effects of luteinizing hormone and follicle stimulating hormone on the developmental competence of porcine preantral follicle oocytes grown *in vitro*. J. Assist. Reprod. Genet..

[15] Shi J.M., Tian X.Z., Zhou G.B., Wang L., Gao C., Zhu S.E., Zeng S.M., Tian J.H., Liu G.S. (2009). Melatonin exists in porcine follicular fluid and improves *in vitro* maturation and parthenogenetic development of porcine oocytes. J. Pineal Res..

[16] Rönnberg L., Kauppila A., Leppäluoto J., Martikainen H., Vakkuri O. (1990). Circadian and seasonal variation in human preovulatory follicular fluid melatonin concentration. J. Clin. Endocrinol. Metab..

[17] Nakamura Y., Tamura H., Takayama H., Kato H. (2003). Increased endogenous level of melatonin in preovulatory human follicles does not directly influence progesterone production. Fertil. Steril..

[18] Diaz E., Pazo D., Esquifino A., Diaz B. (2000). Effects of ageing and exogenous melatonin on pituitary responsiveness to GnRH in rats. J. Reprod. Fertil..

[19] Delgadillo J., Carrillo E., Morán J., Duarte G., Chemineau P., Malpaux B. (2001). Induction of sexual activity of male creole goats in subtropical northern Mexico using long days and melatonin. J. Anim. Sci..

[20] Anand S., Losee-Olson S., Turek F.W., Horton T.H. (2002). Differential regulation of luteinizing hormone and follicle-stimulating hormone in male Siberian hamsters by exposure to females and photoperiod. Endocrinology.

[21] Shiu S.Y., Ng N., Pang S.F. (1996). A molecular perspective of the genetic relationships of G-protein coupled melatonin receptor subtypes. J. Pineal Res..

[22] Reiter R.J. (1991). Pineal melatonin: Cell biology of its synthesis and of its physiological interactions. Endocr. Rev..

[23] Hafez E.S.E. (1952). Studies on the breeding season and reproduction of the ewe Part III. The breeding season and artificial light Part IV. Studies on the reproduction of the ewe Part V. Mating behaviour and pregnancy diagnosis. J. Agric. Sci..

[24] Adam C.L., Atkinson T. (1984). Effect of feeding melatonin to red deer (*Cervus elaphus*) on the onset of the breeding season. J. Reprod. Fertil..

[25] Falcon J., Besseau L., Fazzari D., Attia J., Gaildrat P., Beauchaud M., Boeuf G. (2003). Melatonin modulates secretion of growth hormone and prolactin by trout pituitary glands and cells in culture. Endocrinology.

[26] Jiménez-Ortega V., Barquilla P.C., Pagano E.S., Fernández-Mateos P., Esquifino A.I., Cardinali D.P. (2012). Melatonin supplementation decreases prolactin synthesis and release in rat adenohypophysis: Correlation with anterior pituitary redox state and circadian clock mechanisms. Chronobiol. Int..

[27] Webster J., Barrell G. (1985). Advancement of reproductive activity, seasonal reduction in prolactin secretion and seasonal pelage changes in pubertal red deer hinds (*Cervus elaphus*) subjected to artificially shortened daily photoperiod or daily melatonin treatments. J. Reprod. Fertil..

[28] Itoh M.T., Ishizuka B., Kuribayashi Y., Amemiya A., Sumi Y. (1999). Melatonin, its precursors, and synthesizing enzyme activities in the human ovary. Mol. Hum. Reprod..

